# Day-to-day variations in sleep quality affect standing balance in healthy adults

**DOI:** 10.1038/s41598-018-36053-4

**Published:** 2018-11-30

**Authors:** Luis Montesinos, Rossana Castaldo, Francesco P. Cappuccio, Leandro Pecchia

**Affiliations:** 1 School of Engineering, University of Warwick, Coventry, United Kingdom; 2 Escuela de Ingenieria y Ciencias, Tecnologico de Monterrey, Mexico City, Mexico; 3 Institute of Advanced Study, University of Warwick, Coventry, United Kingdom; 4 Warwick Medical School, University of Warwick, Coventry, United Kingdom; 5 University Hospitals Coventry & Warwickshire NHS Trust, Coventry, United Kingdom

## Abstract

Acute sleep deprivation is known to affect human balance and posture control. However, the effects of variations in sleep quality and pattern over consecutive days have received less attention. This study investigated the associations between day-to-day variations in sleep quality and standing balance in healthy subjects. Twenty volunteers (12 females and 8 males; age: 28.8 ± 5.7 years, body mass index: 23.4 ± 3.4 kg/m^2^, resting heart rate: 63.1 ± 8.7 bpm) with no history of sleep disorders or balance impairments participated in the study. Sleep and balance were assessed over two consecutive days. Sleep quality variations were assessed using sleep diary, actigraphy and heart rate variability (HRV) measures. Sleep was monitored at home, using an unobtrusive wearable device. Balance was assessed in a gait lab using foot centre of pressure (COP) displacement during quiet standing. Subjects with a day-to-day deterioration in sleep quantity and quality (i.e., decreased duration and increased fragmentation, increased nocturnal activity and decreased HRV) exhibited significant changes in balance (i.e., larger COP area, amplitude and standard deviation). Conversely, subjects with no significant alterations in sleep quantity and quality showed no significant changes in COP displacements. These results confirmed our hypothesis that changes in sleep quality and pattern over consecutive days may affect balance.

## Introduction

Human balance is a fundamental skill required to perform most of our daily-life activities. It is the result of the complex integration of the central nervous system with the visual, the vestibular, the proprioceptive and the musculoskeletal systems. During quiet standing, human balance is achieved by constantly reconfiguring ground reaction forces under the feet to counteract the sway of the body. The point of application of the vertical ground reaction force vector is known as centre of pressure (COP)^1^. COP displacement is one of the most common techniques to measure human balance during quiet standing (a technique known as posturography or stabilometry)^2–5^. COP displacement is measured with a force platform or other instrumented surface (e.g. a mat) which produces a two-dimensional time-series representing the COP trajectory in the anterior-posterior (AP) and medial-lateral (ML) axes. COP displacements can be characterised by computing their area, amplitude and variability (i.e., standard deviation), among many other measures. Interestingly, these measures reflect different postural control states. For instance, a wider and more fluctuating COP displacement increases the likelihood of crossing the stability limits, and it is therefore interpreted as a more unstable state. In contrast, a COP displacement that is too narrow and having low variability may indicate an overly stiff postural control (known as “freezing”) that is likely to reduce the ability to adjust to external challenges.

Acute sleep deprivation is associated with alterations in posture control during quiet standing (known as static balance)^6–15^. Balance deficits after intervals of 24 to 48 hours of sleep deprivation are reflected by wider^7,10,13–15^, more fluctuating^6,9,11,12^ and faster^8^ COP displacements in the AP axis. Moreover, vision plays a substantial role in static balance after 24-h sleep deprivation, as suggested by wider and more fluctuating COP displacements observed when subjects are tested with eyes closed than when they are tested with eyes open^12^. After 26 hours of sleep deprivation, subjects also showed higher body sway under the single-task condition and lower body sway under the dual-task condition, suggesting that cognitive load also plays an important role in balance control under sleep deprivation. These findings suggest that the effects of sleep deprivation on postural steadiness found under no cognitive load are compensated with a “freezing” strategy under cognitive load condition^14^. Moreover, older adults (~60 years old) suffer more sleep deprivation effects on balance than young adults (~25 years old)^15^. This finding may be relevant in the context of fall prevention in senior citizens, especially in hospitalised older adults. Therefore, all these studies agreed that long periods of sleep deprivation (≥24 h) are associated with deteriorations in static balance, especially in senior subjects.

More recently, the effects of chronic sleep restriction due to sleep debt and social jet lag have been studied^16,17^. Chronic low sleep quality (i.e. higher sleep fragmentation and lower sleep efficiency) was found to affect balance control causing higher postural instability^16^. Moreover, social jetlag (i.e. the misalignment of the biological driven and the socially dictated sleep times) was also found to deteriorate balance control^17^, as suggested by posture control performance being consistently better on Mondays (after two of days of higher-quality sleep) than on Fridays (after a week of restricted sleep).

Our study investigated the associations between day-to-day variations of sleep quality and static balance. In other words, we focused on the sensitivity of human postural steadiness to shifts in sleep quality over two consecutive nights. The underlying hypothesis of this study was that static balance can be affected by day-to-day variations in sleep quality, and in particular that a deterioration in sleep quality over two consecutive nights would be accompanied by a worsening in postural control. In order to test this hypothesis, a sample of healthy subjects underwent sleep and balance assessment for two consecutive days. Sleep assessment was performed using an unobtrusive wearable device recording electrocardiography and chest actigraphy, as well as a sleep diary. Balance assessment was performed via foot COP displacement using an insole pressure measurement system.

## Methods

### Participants

Participants were recruited using e-mail advertising sent to postgraduate students from the School of Engineering, University of Warwick. Exclusion criteria included history of sleep disorders, neurological or physical disabilities and pharmacological treatment potentially affecting sleep patterns and postural control (e.g. anti-depressants, hypnotics and stimulants).

Baseline characteristics, such as age, height, weight, general health status and use of medications, were collected during a baseline assessment and briefing session. Participants were also asked to complete the Pittsburgh Sleep Quality Index (PSQI) instrument^18^. The PSQI questionnaire provides a global score computed from nineteen self-rated questions related to sleep quality, sleep latency, sleep duration, sleep efficiency, sleep disturbances, use of sleeping medication and daytime dysfunction. The PSQI global score was used to compare baseline sleep quality over the past month between groups.

All subjects provided informed consent prior to participation in the study. This research was performed in accordance with relevant regulations, thus it was approved by the Biomedical and Scientific Research Ethics Committee of the University of Warwick (REGO-2014-1039).

### Equipment

Participants’ monitoring during sleep was performed using the Zephyr BioHarness^TM^ 3.0 (Medtronic, Inc., Annapolis, MD, USA), a patch-type device that measures tri-axial trunk acceleration and one-lead electrocardiogram (ECG) signals at a sampling frequency of 100 Hz and 1 kHz, respectively, and a resolution of 12 bits per sample. The device uses proprietary algorithms to compute user’s activity level and posture based on the acceleration signals. Activity level is reported in gravitational force units (i.e., g-force or simply g, where 1 g = 9.806 m/s^2^) within a range of 0 to 16 g and is computed as $Activity=\sqrt{{x}^{2}+{y}^{2}+{z}^{2}}$, where *x*, *y* and *z* (the vertical, medial-lateral and anterior-posterior axes, respectively) are the averages of the three-axial acceleration magnitudes over the previous 1-second window. Posture is reported in degrees as the angle of deviation from the vertical axis. Activity level and Posture time-series are reported with a frequency of 1 sample per second. Moreover, this device performs R peak detection on the ECG waveform and reports R-R intervals in milliseconds. Raw three-axial accelerations, ECG signals, R-R interval time-series, and a summary file containing the activity and posture time-series are stored in the internal memory of the device during usage and can be downloaded for further processing. The validity and reliability of the Zephyr BioHarness^TM^ have been found to be strong to very strong for heart rate, acceleration and posture monitoring at low to moderate physical activity levels^19,20^.

**Figure 1:**
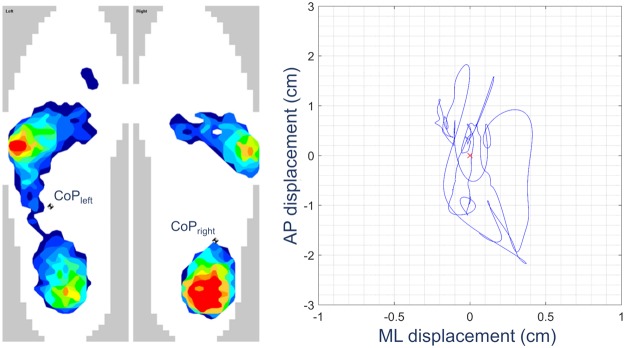
Plantar pressure map and centre of pressure trajectory. Left: Representative bi-plantar pressure map during quiet standing. The black and white circle represents the foot centre of pressure computed from pressure distribution data. Right: Representative centre of pressure trajectory (left foot) for a 20-second window.

Balance testing was performed using the Tekscan® F-Scan® system (Tekscan, Inc., South Boston, MA, USA), a plantar pressure measurement and analysis system. This system is based on a pair of ultra-thin (0.15 mm) instrumented insoles with a spatial resolution of 3.9 pressure-sensing elements per cm^2^. Bi-plantar pressure data were collected at a rate of 200 frames per second. Based on pressure data, the F-Scan Research 7 software computes the foot COP location for each frame. COP displacement is stored as time-series of numerical data in the anterior-posterior (AP) and medial-lateral (ML) axes in relation to the subject’s orientation. Figure 1 shows a typical bi-plantar pressure distribution map during quiet standing and the resulting centre of pressure displacement trajectory. As per the manufacturer’s recommended procedures, the F-Scan® system was calibrated for each participant following the point calibration routine, the suggested method for standing balance trials. This calibration procedure requires each sensor to be individually calibrated by having the subject standing on a single foot at a time for a few seconds (~5 seconds). In fact, Hsiao *et al*. emphasised on the importance of calibrating the system in actual experimental conditions prior to use^21^. Following the proper calibration procedure, the accuracy of the F-Scan® has been found to be satisfactory (i.e., with a measurement error less than 6%) when the sensors are subjected to static loads (e.g. during quiet standing) and the pressure applied during the protocol is comparable with that used during calibration^21^. These considerations are worth mentioning, as some studies have questioned the validity and reliability of the Tekscan® F-Scan® system, when utilised with dynamic loads (i.e. walking^22^) or when the sensors were calibrated using two pressure values and tested over a wider range^23^.

### Study protocol

**Figure 2:**
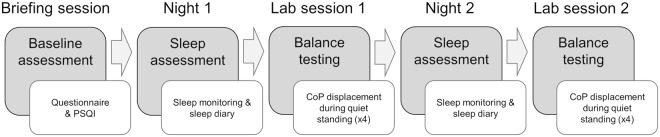
Schematic diagram of the study protocol. Sleep monitoring was performed using a wearable device that records trunk acceleration and electrocardiogram signals. Balance testing was performed using a plantar pressure measurement and analysis system based on a pair of instrumented insoles.

A schematic of the study protocol is shown in Fig. 2. After baseline assessment, participants underwent sleep and balance assessment for two consecutive days. For sleep assessment, they were asked to wear the BioHarness^TM^ during sleep; i.e., to apply it at the time of usual bedtime and to take it off after the final awakening. Additionally, subjects were required to complete the Consensus Sleep Diary^24^ every morning immediately after getting out of bed during their participation in the study. Participants were invited to stick to their regular sleep schedule and habits (i.e. no intervention was applied).

Balance was assessed in two morning sessions starting at the same time of the day (9:00 or 10:00 a.m.) for any given participant. Previous studies have suggested that COP measures of postural control change throughout the day, allegedly following a circadian pattern^7–9^. By starting both sessions at the same time of the day, the influence of time of day on postural control measures was discarded as a potential confounder. At each session, participants were asked to complete four quiet standing trials with eyes open. Namely, they were instructed to stand quietly on the foot pressure sensors with arms hanging naturally at their sides and eyes staring at a fixed point on the wall in front of them. The sensors were attached to the floor side-by-side in a comfortable position for each participant (about shoulder width). The duration of each trial was 30 seconds and a brief resting interval (~15 seconds) was allowed between trials. Participants wore socks but no shoes during the whole duration of the session.

### Data processing

Data collected via the sleep diary, the BioHarness and the Tekscan system were processed as follows in order to compute a set of sleep and balance measures (see Table 1 for a summary of those measures with their definitions).

**Table 1:** Summary of sleep and centre of pressure displacement measures

Measure	Units	Description
**Sleep measures**		
*Sleep diary measures*		
Sleep onset latency (SOL)	min	Duration of the transition interval from wakefulness to sleep.
Wake after sleep onset (WASO)	min	Total duration of intervals of wakefulness between sleep onset and final awakening. It is a measure of sleep fragmentation.
Total sleep time (TST)	h	Total duration of actual sleep
Sleep efficiency (SE)	%	Ratio of total sleep time to time-in-bed expressed as a percentage
Subjective sleep quality (SSQ)	—	Subjective appraisal of quality of sleep (1 = Very poor, 2 = Poor, 3 = Fair, 4 = Good, 5 = Very good)
*Activity level measures*		
ACT_MEAN	counts	Mean activity counts per epoch for the whole sleep opportunity
ACT_SD	counts	Standard deviation of activity counts per epoch for the whole sleep opportunity
Activity Index (AI)	%	Ratio of the number of epochs with activity counts > 0 to the total number of epochs expressed as a percentage
Fragmentation Index (FI)	%	Ratio of inactive intervals (activity counts = 0) with duration ≤ 5 minutes to the total number of inactive intervals expressed as a percentage
MAX_REST	min	Duration of the longest inactive interval (activity counts = 0)
AVG_REST	min	Average duration of all inactive intervals (activity counts = 0)
*HRV measures*		
LF	ms^2^	Power in the low frequency range (0.04–0.15 Hz)
HF	ms^2^	Power in the high frequency range (0.15–0.4 Hz)
LF normalised	—	LF power in normalised units, computed as LF/LF+HF × 100
HF normalised	—	HF power in normalised units, computed as HF/LF+HF × 100
LF/HF ratio	—	Ratio LF to HF
Approximate entropy (ApEn)	—	A measure of the regularity or predictability of fluctuations in a time-series
Sample entropy (SampEn)	—	A modification of ApEn that reduces the chances of overestimating the entropy in a time-series
**COP displacement measures**		
Area	cm^2^	Area of the ellipse that contains 95% of the COP points
Amplitude	cm	Distance between the minimum and maximum positions. Also known as “range”.
Standard deviation	cm	Dispersion of the COP position around the mean

HRV = Heart rate variability.

COP = Centre of pressure.

#### Sleep Diary Measures

Five sleep measures were extracted from the sleep diary: (1) sleep onset latency (SOL); (2) wake after sleep onset (WASO), a measure of sleep fragmentation; (3) total sleep time (TST) or sleep duration; (4) sleep efficiency (SE), and; (5) subjective sleep quality (SSQ).

#### Sleep Activity Level Measures

Activity level signals were processed to compute six measures of activity during sleep. Firstly, raw signals were trimmed using posture data to discard intervals outside the sleep period (i.e. before getting into and after getting out of bed). Subsequently, the signals were segmented into continuous, non-overlapping 1-minute epochs and activity counts were computed for each epoch using the zero-crossing mode; i.e., the activity level was compared with the reference activity level, and each threshold crossing generated an activity count^25^. The threshold was set to 0.1 g for high sensitivity. Finally, the following activity measures were computed from the activity counts: mean and standard deviation of activity counts per epoch (ACT_MEAN and ACT_SD, respectively), activity index (AI), fragmentation index (FI), and maximum and average duration of the inactive intervals (MAX_REST and AVG_REST, respectively). These measures were computed using in-house written scripts in Matlab R2016b (The Mathworks, Inc., Natick, MA, USA).

#### Heart Rate Variability Measures

Heart rate variability (HRV) measures were computed from R-R series in order to characterise autonomic cardiac modulation during sleep. A higher parasympathetic tone has been observed during non-rapid eye movement sleep (NREM), particularly during deep sleep; in contrast, a higher sympathetic tone has been observed during wake intervals, rapid eye movement sleep (REM) and sleep arousals^26^. Therefore, the HRV analysis provided with an indication of the presence of wake intervals and arousals, as well as of shorter deep sleep periods.

Firstly, the R-R series were trimmed using posture data to discard heart beats outside the sleep period. Subsequently, the software HRVanalysis^27^ was used to compute four HRV measures: two frequency-domain measures (LF and HF power) and two nonlinear measures (Approximate entropy and Sample entropy). The algorithms for anomalous R-R peaks exclusion and correction implemented in the HRVanalysis software were applied. Additionally, three frequency-domain measures (LF normalised, HF normalised and LF/HF ratio) were computed using in-house written scripts in Matlab R2016b. The meaning of these HRV measures has been widely described in detail elsewhere^28,29^. In the context of sleep assessment, those features are associated with specific sleep stages and relevant phenomena (e.g. arousals)^26^. In the frequency-domain, HF power describes the parasympathetic activity, whereas LF power describes both parasympathetic and sympathetic activity. Thus, the relationship between both branches is normally explored with the normalised frequency values and the LF/HF ratio. Finally, entropy measures represent an index of complexity in the cardiac signal. An increase in complexity (i.e., an increase in the entropy measure) is associated with parasympathetic modulation and its decrease is interpreted as the result of an increased sympathetic tone.

#### Balance Measures

COP time-series were trimmed to discard the initial and last 5 seconds of each trial in order to account for the “adaptation phase” of the participant to the quiet standing task and for the effects of fatigue or lack of attention associated to a sustained task, respectively^5^. Subsequently, the COP time-series were passed through a fourth-order zero-phase Butterworth low-pass digital filter with a cut-off frequency of 5 Hz in order to remove acquisition noise. Afterwards, they were detrended (i.e., the mean was subtracted). Hence, the analysis of the COP displacement was carried out relative to its mean position and not to the origin of the sensor’s coordinate system. Finally, three COP displacement measures were computed as described in detail by Duarte *et al*.^2^: Area, Amplitude and Standard deviation. These measures were computed for left and right feet independently. Additionally, the measures for left and right feet were averaged. Amplitude and Standard deviation were computed in the AP axis only, as previous studies have shown that it is mainly on this axis that balance alterations are observed^10,12,14,15^. Scripts for COP data processing were also written in Matlab R2016b.

The datasets generated during the current study are available from the corresponding author on reasonable request.

### Statistical analysis

Participants were stratified according to the sleep quality scores they reported in the sleep diary (SSQ) in Control group (i.e., participants who reported no variation in sleep quality over two consecutive nights) and Case group (i.e., participants who reported a variation in sleep quality over two consecutive nights; e.g. good sleep quality in one night and poor sleep quality in the other). The validity of self-reported sleep quality was tested by running pairwise comparisons for all other sleep measures within each group. By definition, no differences over consecutive nights were expected for the Control group, while significant differences were expected for the Case group. Two-sided Wilcoxon paired tests with a significance level set at 0.05 were used for these comparisons, given that most sleep measures exhibited a non-normal distribution (Shapiro-Wilk test with a p-value < 0.05).

Subsequently, a repeated measures ANOVA-type rank test for factorial designs was performed in order to test the main effects and the interaction effects of Group and Session on balance measures^30^. This test was developed for experimental designs where subjects are stratified in several groups, as well as observed at different time points (i.e., mixed designs). Importantly, these tests have been found to be robust with respect to outliers and small sample sizes. The computational implementation of this test provided by the authors through the R package nparLD version 2.1 was used^31^. The main effects and interaction effects of Group and Session were tested for all balance measures. A p-value < 0.05 was accepted as indicative of statistical significance. This analysis was performed in R version 3.4.1 (R Foundation for Statistical Computing, Vienna, Austria).

Finally, differences in balance measures between sessions were investigated for each group (i.e. post hoc comparisons): for the Control group, pairwise comparisons were always made between Session 2 and Session 1, given that by definition for this group sleep quality was equally rated in both sleep opportunities; for the Case group, pairwise comparisons were done between the session with the poorest sleep quality and the session with the best sleep quality, regardless of the order in which they were chronologically presented. Two-tailed Wilcoxon paired tests were performed given the non-normal distribution of most balance measures (Shapiro-Wilk test with a p-value < 0.05). A p-value < 0.05 was accepted as indicative of statistical significance. These tests were conducted in Matlab R2016b.

## Results

### Participants’ baseline characteristics and stratification

Twenty healthy volunteers (12 females and 8 males) participated in our study. The sample had an overall mean (standard deviation) age of 28.8 (5.7) years, height of 170.8 (8.3) cm, mass of 68.7 (13.2) kg, body mass index of 23.4 (3.4) kg/m^2^, heart rate at rest of 63.1 (8.7) beats/minute, PSQI score of 5.1 (2.4) and sleep duration of 7 (1) hours during the past month. No significant differences were found between groups for these characteristics (Table 2).

**Table 2:** Baseline characteristics of study participants.

Variable	All (N = 20)		Control group (N = 6)		Case group (N = 14)		p-value
	Mean	SD	Mean	SD	Mean	SD	
Age (years)	28.8	5.7	29.5	5.7	28.4	5.9	0.711
Mass (kg)	68.7	13.2	64.3	11.3	70.6	13.9	0.339
Height (cm)	170.8	8.3	167.6	6.8	172.1	8.8	0.279
BMI (kg/m^2^)	23.4	3.4	22.8	3.7	23.7	3.4	0.608
HR (bpm)	63.1	8.7	63.4	8.2	63.0	9.2	0.925
PSQI	5.1	2.4	5.0	1.7	5.1	2.7	0.908
TST (hours)	7.0	1.0	7.2	1.0	7.0	1.1	0.727

Mean and standard deviation for all subjects, subjects without day-to-day variation in sleep quality (Control group) and subjects with variation in day-to-day sleep quality (Case group). P-values from two-tailed paired t-tests are also shown.

SD = Standard deviation.

BMI = Body Mass Index.

HR = Heart Rate at rest.

PSQI = Pittsburgh Sleep Quality Index.

TST = Total sleep time for the past month.

Six participants reported no variation in sleep quality over two consecutive nights (Control group), whereas 14 participants reported a variation in sleep quality over two consecutive nights (Case group). No significant differences were found in sleep measures over the two consecutive nights for the Control group. Conversely, the Case group exhibited significant differences for some sleep measures (Table 3). Namely, for the poorest sleep quality night (i.e., the lowest-rated) the Case group exhibited:

- Longer wake-after-sleep-onset (p = 0.043) and shorter total sleep time (p = 0.038), as self-reported in the sleep diary.
- Higher mean and standard deviation of activity counts per epoch (p = 0.033 and p = 0.048, respectively), higher activity index (p = 0.033) and shorter mean duration of the longest inactive interval (p = 0.041) as computed from the body acceleration signals.
- Lower heart rate variability, as reflected by lower power in the high-frequency band (p = 0.033) and lower approximate and sample entropies (p = 0.021 and p = 0.006, respectively).

**Table 3:** Day-to-day differences in sleep measures.

Measure	Control group (N = 6)			Case group (N = 14)			
	MD	IQR	p	MD	IQR	p	Trend
*Sleep diary measures*							
SOL (min)	−7.5	20	0.656	2.5	17	0.386	↑
WASO (min)	−0.5	5	0.375	4	10	**0.043**	↑↑
TST (h)	−0.5	1.5	0.250	−0.675	1.83	**0.038**	↓↓
SE (%)	2.5	5	0.438	−1.5	10	0.236	↓
*Activity level measures*							
ACT_MEAN (counts)	−0.045	0.097	0.438	0.024	0.109	**0.033**	↑↑
ACT_SD (counts)	−0.045	0.227	0.219	0.075	0.266	**0.048**	↑↑
Activity Index (%)	−1.514	4.978	0.313	0.793	4.149	**0.033**	↑↑
Fragmentation Index (%)	−3.199	12.433	0.156	3.571	13.166	0.735	↑
MAX_REST (min)	−13	30	0.219	−9	22	**0.041**	↓↓
AVG_REST (min)	1.575	6.764	0.844	−1.416	4.49	0.191	↓
*HRV measures*							
LF (ms^2^)	52.556	767.843	0.438	−238.447	615.061	0.146	↓
HF (ms^2^)	−44.143	231.888	1	−115.617	241.026	**0.033**	↓↓
LF normalised	0.689	6.171	1	2.323	8.165	0.127	↑
HF normalised	−0.689	6.171	1	−2.323	8.165	0.127	↓
LF/HF ratio	0.188	1.095	0.844	0.323	1.745	0.191	↑
ApEn	−0.001	0.05	1	−0.037	0.059	**0.021**	↓↓
SampEn	0.034	0.149	0.844	−0.042	0.109	**0.006**	↓↓

Median difference and interquartile range of the median difference subjects without day-to-day variation in sleep quality (Control group) and subjects with variation in day-to-day sleep quality (Case group). P-values from two-tailed paired Wilcoxon tests are also shown.

MD = Median difference; IQR = Interquartile range.

p = P-values from two-tailed paired Wilcoxon tests. Bold values indicate significant differences (p < 0.05).

↓↓ (↑↑) = Significantly lower (higher) for poorer sleep quality night.

↓ (↑) = Lower (higher) for poorer sleep quality night.

SOL = Sleep onset latency; WASO = Wake after sleep onset; TST = Total sleep time; SE = Sleep efficiency.

HRV = Heart rate variability.

### Group and Session main effects and interaction effects on balance measures

The main effects of Group and Session were not significant for any COP displacement measure (Table 4). However, two COP displacement measures showed significant Group*Session interaction effects:

- Area of displacement for the right foot (p = 0.025).
- Standard deviation (AP axis) for the right foot (p = 0.017).

**Table 4:** Main effects and interactions effects of Group and Session on balance measures.

Factor/Interaction	Group		Session		Group*Session	
Measure	F_n_	p	F_n_	p	F_n_	p
Area, left foot (cm^2^)	0.096	0.757	1.427	0.232	0.603	0.437
Area, right foot (cm^2^)	0.086	0.770	0.029	0.866	5.027	**0.025**
Area, average (cm^2^)	0.062	0.804	0.31	0.578	0.734	0.392
Amplitude, AP, left foot (cm)	0.118	0.731	0.982	0.322	0.271	0.603
Amplitude, AP, right foot (cm)	0.242	0.623	1.867	0.172	3.243	0.072
Amplitude, AP, average (cm)	0.074	0.786	3.652	0.056	0.989	0.32
Standard deviation, AP, left foot (cm)	0.032	0.859	0.436	0.509	0.901	0.342
Standard deviation, AP, right foot (cm)	0.211	0.646	0.002	0.962	5.656	**0.017**
Standard deviation, AP, average (cm)	0.049	0.824	0.637	0.425	2.550	0.11

AP = Anterior-posterior.

Fn = ANOVA-type statistic.

p = P-values from ANOVA-type non-parametric two-tailed paired tests. Bold values indicate significant differences (p < 0.05).

### Pairwise comparisons for balance measures

As reported in Table 5, eight COP displacement measures exhibited significant differences after sleep deterioration (Case group). Namely, after the lowest-rated sleep participants showed a less stable balance as reflected by:

- An increase in the area of displacement for left and right feet, as well as for the averaged measure (p = 0.049, p = 0.011 and p = 0.035, respectively).
- An increase in the amplitude of displacement (AP axis) for left and right feet, as well as for the averaged measure (p = 0.025, p = 0.013 and p = 0.020, respectively).
- An increase in standard deviation (AP axis) for the right foot and the average for both feet (p = 0.035 and p = 0.042, respectively).

**Table 5:** Day-to-day differences in centre of pressure displacement measures.

Measure	Control group (N = 6)			Case group (N = 14)			
	MD	IQR	p	MD	IQR	p	Trend
Area, left foot (cm^2^)	0.014	0.248	0.688	0.048	0.281	**0.049**	↑↑
Area, right foot (cm^2^)	−0.049	0.207	0.313	0.031	0.176	**0.011**	↑↑
Area, average (cm^2^)	0.003	0.55	0.844	0.042	0.239	**0.035**	↑↑
Amplitude, AP, left foot (cm)	0.248	0.437	0.563	0.319	1.693	**0.025**	↑↑
Amplitude, AP, right foot (cm)	−0.066	0.969	0.844	0.228	0.609	**0.013**	↑↑
Amplitude, AP, average (cm)	0.091	0.343	0.688	0.252	1.121	**0.020**	↑↑
Standard deviation, AP, left foot (cm)	0.006	0.099	0.844	0.056	0.364	0.058	↑
Standard deviation, AP, right foot (cm)	−0.032	0.089	0.438	0.046	0.217	**0.035**	↑↑
Standard deviation, AP, average (cm)	0.005	0.12	1	0.048	0.283	**0.042**	↑↑

Median difference and its interquartile range for subjects without day-to-day variation in sleep quality (Control group) and subjects with variation in day-to-day sleep quality (Case group).

AP = Anterior-posterior.

MD = Median difference; IQR = Interquartile range.

p = P-values from two-tailed paired Wilcoxon tests. Bold values indicate significant differences (p < 0.05).

↓↓ (↑↑) = Significantly lower (higher) after poorer sleep quality night.

↓ (↑) = Lower (higher) after poorer sleep quality night.

**Figure 3:**
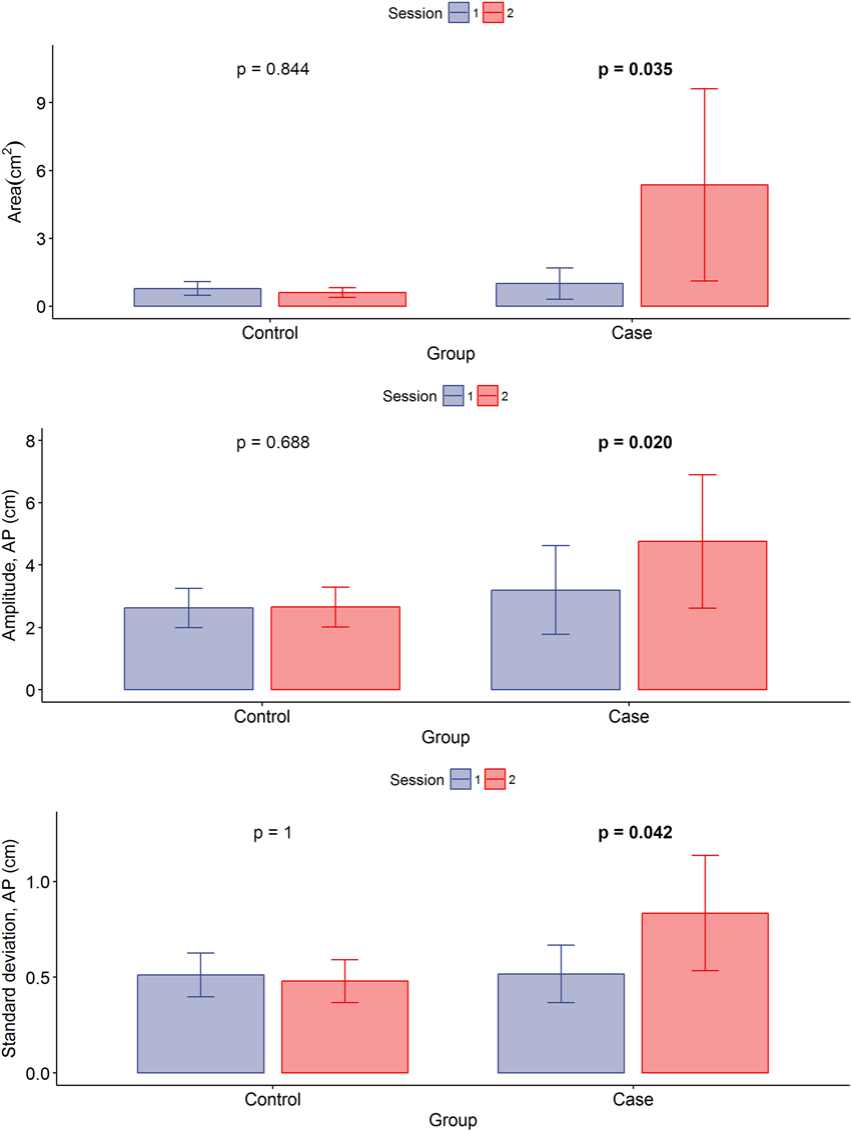
Centre of pressure displacement measures. Mean (bars) and standard error of the mean (error lines) by group and session. The Control group comprises subjects without day-to-day variations in sleep quality; the Case group comprises subjects with variations. AP = Anterior-posterior; p = p-value from two-tailed paired Wilcoxon tests.

Conversely, no significant COP displacement measure variations were observed in the Control group (i.e., subjects presenting no sleep quality variations). Figure 3 illustrates the observed results for the feet-averaged COP displacement measures.

## Discussion

This study investigated the associations between day-to-day variations in sleep quality and balance during quiet standing. We hypothesised that postural steadiness, measured by foot COP displacement, may be affected by changes in sleep quality over two consecutive nights and that deterioration in sleep quality impairs balance control. Firstly, we wanted to explore whether or not day-to-day self-reported sleep quality was confirmed by instrumented sleep assessment. Therefore, participants were stratified in two groups based on whether or not they reported a shift in sleep quality over two consecutive nights. Importantly, reported changes in sleep quality were not artificially induced; they were rather the consequence of spontaneous sleep disturbances experienced during the lowest-rated sleep opportunity (e.g. the need to use the bathroom, an uncomfortable room temperature and involuntarily waking up in the middle of the night or early in the morning for no apparent reason, among the most referred disturbances). Subjects reporting a shift in sleep quality reported significantly higher sleep fragmentation (WASO) and significantly lower sleep duration (TST) for the lowest-rated sleep opportunity. They also showed higher levels of activity and shorter inactive intervals as measured via body acceleration signals, suggesting a less quiet and more fragmented sleep. These results suggest that self-reported sleep quality was indeed associated with more discontinuous and less quiet sleep, in line with the study by Furtado *et al*.^16^, in which higher WASO and activity levels were observed in subjects with low-quality sleep over one week. Moreover, in our study, subjects reporting a variation in sleep quality also exhibited higher sympathetic activity (i.e., lower heart rate variability) during the sleep opportunity, which according to existing literature suggests the presence of more wake intervals and/or arousals, and fewer and/or shorter deep sleep intervals^26^. All these differences confirmed that the subjective sleep quality appraisal that participants made via the sleep diary reflected actual variations in objective sleep measures.

The main effects and the interaction effects of Group and Session on balance measures were tested. No significant Group or Session main effects were found for any COP measure, confirming the overall homogeneity in balance performance between groups and sessions. Conversely, two COP displacement measures more showed significant Group*Session interactions, confirming our hypothesis that day-to-day variations in balance are associated with variations in sleep quality over consecutive nights.

The Group*Session interaction effects abovementioned were found to be attributable to subjects that exhibited a variation in sleep quality over consecutive nights. The group of subjects reporting and exhibiting worsening in sleep measures over two consecutive days also exhibited larger COP displacements (i.e., amplitude and area) and fluctuations (i.e., standard deviation), particularly in the anterior-posterior axis. These results are in line with previous studies, which have also found larger, more fluctuating and faster COP displacements in the anterior-posterior axis as a result of 24 to 48 hours of sleep deprivation^6–15^. This suggests that the alterations in postural control observed after a day-to-day deterioration in sleep quality have similar manifestations (direction) to those of the ones produced by longer periods of sleep deprivation. These alterations are expected to increase (magnitude) in older adult populations, as has been found in previous studies^15^.

Altogether, our results confirm that day-to-day variations in sleep quality are associated with variations in static balance. The fact that no differences were found in the group of participants that reported and exhibited no differences in sleep quality over two nights supports this conclusion.

The neurophysiological mechanisms behind the observed alterations in postural control need to be elucidated. It is known that both vigilant attention and the visual system are affected by sleep deprivation^32–35^. Both have also been found to play an important role in postural control^36–40^. Future studies could further investigate the effects of day-to-day variations in sleep quality and standing balance by observing its modulation by available attentional resources (e.g. cognitive plus postural task versus only postural task) and/or visual conditions (e.g. eyes open versus eyes closed).

Although the effects of acute total sleep deprivation^6–15^, chronic low sleep quality^16^ and social jetlag^17^ on postural control had been previously investigated, to the best of our knowledge, the novelty of our paper is that it focused on whether or not spontaneous variations in sleep quality over two consecutive nights may affect static balance. Our findings suggest that a deterioration in sleep quality over two consecutive nights is associated with balance during quiet standing, as measured by the centre of pressure displacement.

This finding may be relevant in the context of fall prevention, as previous studies have found significant associations between COP displacement measures and risk of falling (although a consensus has not yet been reached on what are the key balance outcome measures for fall prediction)^41–44^. Therefore, this topic warrants more studies to produce additional evidence.

The present study has some limitations. Centre of pressure displacement is the simplest model of human balance, thus it reflects only partially human postural steadiness and limits its interpretation^45^. The adoption of more advanced models and the introduction of additional testing conditions (e.g. under different cognitive loads and visual conditions) in further studies could give deeper insights on which mechanisms of balance control are more affected by sleep deterioration.
